# Stromal Interferon-γ Signaling and Cross-Presentation Are Required to Eliminate Antigen-Loss Variants of B Cell Lymphomas in Mice

**DOI:** 10.1371/journal.pone.0034552

**Published:** 2012-03-30

**Authors:** Armin Gerbitz, Madhusudhanan Sukumar, Florian Helm, Andrea Wilke, Christian Friese, Cornelia Fahrenwaldt, Frank M. Lehmann, Christoph Loddenkemper, Thomas Kammertoens, Josef Mautner, Clemens A. Schmitt, Thomas Blankenstein, Georg W. Bornkamm

**Affiliations:** 1 Department of Immunology, Charité Berlin, Berlin, Germany; 2 Institute of Clinical Molecular Biology and Tumor Genetics, Helmholtz Center Munich, Munich, Germany; 3 Department of Pathology, Charité Berlin, Berlin, Germany; 4 Department of Pediatrics, Technical University (TU) Munich, Munich, Germany; 5 Clinical Cooperation Group Pediatric Tumor Immunology, TU Munich and Helmholtz Center Munich, Munich, Germany; 6 Department of Hematology and Oncology, University of Erlangen, Erlangen, Germany; 7 Department of Hematology and Oncology, Charite Berlin, Berlin, Germany; 8 Max-Delbrück-Center for Molecular Medicine, Berlin, Germany; University of Nebraska – Lincoln, United States of America

## Abstract

To study mechanisms of T cell-mediated rejection of B cell lymphomas, we developed a murine lymphoma model wherein three potential rejection antigens, human c-MYC, chicken ovalbumin (OVA), and GFP are expressed. After transfer into wild-type mice 60–70% of systemically growing lymphomas expressing all three antigens were rejected; lymphomas expressing only human c-MYC protein were not rejected. OVA expressing lymphomas were infiltrated by T cells, showed MHC class I and II upregulation, and lost antigen expression, indicating immune escape. In contrast to wild-type recipients, 80–100% of STAT1-, IFN-γ-, or IFN-γ receptor-deficient recipients died of lymphoma, indicating that host IFN-γ signaling is critical for rejection. Lymphomas arising in IFN-γ- and IFN-γ-receptor-deficient mice had invariably lost antigen expression, suggesting that poor overall survival of these recipients was due to inefficient elimination of antigen-negative lymphoma variants. Antigen-dependent eradication of lymphoma cells in wild-type animals was dependent on cross-presentation of antigen by cells of the tumor stroma. These findings provide first evidence for an important role of the tumor stroma in T cell-mediated control of hematologic neoplasias and highlight the importance of incorporating stroma-targeting strategies into future immunotherapeutic approaches.

## Introduction

Tumor cells harbor genetic changes that frequently cause the synthesis of mutated proteins. The ability of the immune system to recognize small genetic changes including point mutations has created great hopes for cancer treatment. Mutated proteins that may serve as targets for T cell rejection are regularly found in human tumors and in murine tumor models, particularly those induced by physical or chemical carcinogens [1]–[3]. Unfortunately no generic immunogenic mutations have been found that might be used to raise a neutralizing immune response against a given tumor type and foreign antigens are usually not available except in some virus-associated tumors. Most attempts of immunotherapy have therefore targeted auto-antigens preferentially expressed by the tumor. Usually, only low-affinity T cells with limited therapeutic potential against these antigens are systemically present since these must evade negative selection in the thymus [4], [5].

The ability of the immune system to fight hematologic malignancies efficiently has been demonstrated in two paradigmatic clinical settings in humans: allogeneic stem cell transplantation (SCT) for treatment of chronic myeloid leukemia (CML) [6], [7] and adoptive T cell therapy (ATCT) for the treatment of Epstein-Barr virus-induced post transplant lymphoproliferative disease (PTLD) [8]–[10]. Both have in common that T cells target foreign antigens: minor histocompatibility antigens in the case of CML and viral antigens in PTLD. This underscores the notion that cancer immunotherapy should not rely on a negatively selected T cell repertoire.

The incidence of high-grade B cell lymphomas has increased over the last decades in western countries for unclear reasons [11]. Improvement of conventional chemotherapy regimens translated into increased 5-year survival rates (currently 60% for all B cell lymphoma entities) [12], [13]. Relapse of aggressive B cell lymphomas after chemotherapy remains to be a difficult clinical issue and allogeneic SCT is frequently the last treatment option. Contrary to CML, the benefit of allogeneic SCT for treatment of high-grade lymphomas is not well established. Several studies suggested a potential graft-versus-leukemia/lymphoma (GvL) effect for acute lymphoblastic leukemia (ALL) and several types of non-Hodgkin lymphomas (NHL) [14]–[16], but comparison of different trials could not establish a GvL effect unequivocally for diffuse large B cell lymphomas (DLBCL) and Burkitt's lymphoma (BL) [17].

Over the last years, it became evident that immunotherapy against solid tumors is not effective in the long term when only antigen-expressing tumor cells are targeted. To eliminate antigen-negative tumor cells as well, targeting the tumor stroma is evidently important and any effective T cell therapy has to include activity against stromal tissue. In solid tumors the term stroma refers to non malignant cells surrounding and potentially supporting malignant growth including vessles, connective tissue, but also hematopoietic cells such as macrophages or other antigen presenting cells. For example, outgrowth of antigen-loss variants of carcinogen-induced sarcomas is prevented by antigen-specific T cells that eradicate antigen cross-presenting stroma cells in an IFN-γ-dependent manner [18]–[21]. In contrast, the role of the stroma in aggressive B cell lymphomas is ill-defined and it is unclear, whether conclusions drawn from the analysis of solid tumors hold true for hematopoietic malignancies. We have established a lymphoma model to investigate mechanisms of antigen-dependent rejection in hematopoietic tumors. Ovalbumin (OVA) and/or green fluorescence protein (GFP) were expressed as tumor-specific foreign antigens in lymphomas derived from λ-c-myc transgenic mice [22]. Using this model, we addressed whether IFN-γ signaling in the tumor or in the host influences rejection of antigen-expressing lymphoma cells, rejection of antigen-loss variants, and overall survival. Here, we demonstrate that the stromal IFN-γ signaling-dependent mechanism known to contribute to solid tumor rejection, is also an essential component of immune-mediated B cell lymphoma elimination.

## Results

### Expression of OVA and/or GFP leads to lymphoma rejection in immunocompetent hosts

**Figure 1:**
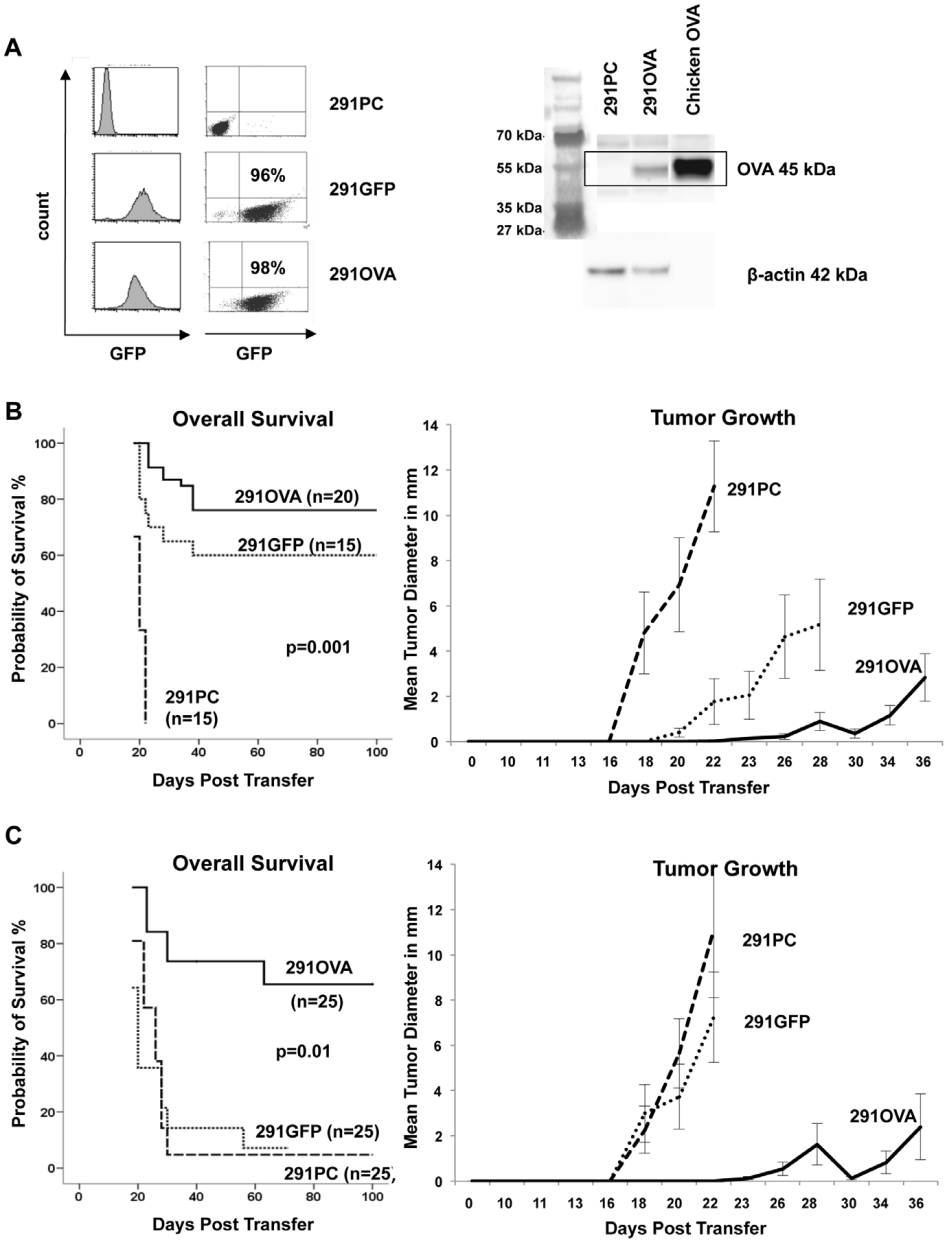
OVA and GFP serve as foreign antigens and mediate rejection of the λ-huMYC lymphoma cell line 291 in wild-type mice. (A) 291PC cells were transduced with retroviruses expressing IRES-GFP or OVA-IRES-GFP. Fluorescence activated cell sorting for GFP resulted in a purity of 96 to 98% GFP-positive cells, respectively (left panel of A). Expression of OVA was confirmed by Western blot analysis (A, right panel). (B and C) 1×10^5^ λ-huMYC 291PC (parental cells) lymphoma cells (dashed line), retrovirally transduced with IRES-GFP (291GFP) (dotted line), or OVA-IRES-GFP (291OVA) (solid line) were injected s.c. into either wild-type (B) or into GFP-transgenic UBI-GFPtg mice (C). Overall survival (left panels) and tumor growth (right panels) were monitored as described in Material & Methods (data are compiled from 3 independent experiments).

To generate lymphoma cells that express defined foreign antigens, a cell line (291PC, parental cells) was established from a spontaneous λ-hu-MYC lymphoma and transduced with retroviruses encoding either OVA cDNA upstream of IRES-GFP (termed 291OVA) or IRES-GFP as control (termed 291GFP). More than 95% of the sorted cells expressed GFP (Figure 1A, left panel). Expression of OVA in transduced cells was confirmed by Western blotting (Figure 1A, right panel). When inoculated into wild-type mice, 291OVA cells thus express OVA, GFP and human c-MYC, 291GFP cells GFP and c-MYC, and untransduced 291PC cells c-MYC as foreign antigen. The human and murine c-MYC proteins, however, share more than 90% amino acid sequence identity. To address whether the human c-MYC protein acts as a rejection antigen, increasing cell numbers of the lymphoma cell line 291PC were injected subcutaneously into wild-type (Figure 1B) and GFP-transgenic mice (Figure 1C). Injection of 1×10^5^ cells or more led to 100% mortality. When less than 1×10^5^ cells were inoculated, more than 10% of the animals survived for 100 days. Subcutaneous injection resulted in local lymphoma growth, but in almost all cases systemic disease was also observed with lymphomas arising in the spleen. Thus, MYC lymphoma cells are capable of tumor-initiation and are not eliminated by rejection if more than 1×10^5^ cells are transferred.

**Figure 2:**
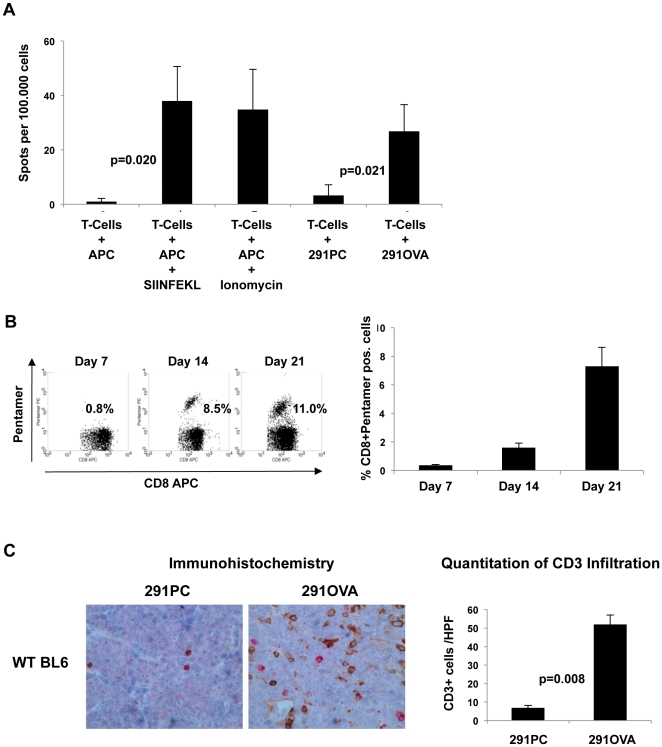
Inoculation of OVA-expressing lymphoma cells into wild-type mice elicits an OVA-specific CD8+ T cell response. (A) 1×10^5^ CD90-selected splenic T cells of mice challenged with 291OVA cells were restimulated with peritoneal macrophages (APC, antigen presenting cells) in the presence or absence of SIINFEKL peptide (p = 0.020) or with 291 parental (291PC) or OVA-expressing lymphoma cells (291OVA, p = 0.021, Mann-Whitney test, n = 5 animals/group). (B) T cell responses against OVA were monitored at different time points by flow cytometry using SIINFEKL-specific pentamers in the peripheral blood of wild-type mice inoculated with 291OVA cells. The percentage of SIINFEKL pentamer-positive CD8+ T cells increased over time as shown for one representative mouse (B, left panel). Mean values (+/− standard deviation) of percentage of SIINFEKL pentamer-positive CD8+ T cells increased over time as compiled from 5 mice (B, right panel). (C) After inoculation of 1×10^5^ 291 parental or 291OVA cells, the developing lymphomas were analyzed by immunohistochemistry for infiltration of CD3-positive (peroxidase brown staining) and perforin expressing (alkaline phosphatase red staining) cells (C, left panel). CD3− and perforin-positive cells were quantified in blinded fashion by counting 10 high power fields (HPF, 400×) per section. Values are given as a mean ± standard error of the mean (C, right panel). Only CD3-positive and CD3/perforin-double positive cells (CD3/HPF) were regarded as T cells, whereas single perforin-positive cells were considered to be NK cells and excluded from the analysis. For each group 8–15 sections were analyzed. Mann Whitney-U test was used for comparison.

Next, we addressed whether lymphomas expressing OVA and GFP are rejected in wild-type mice. Injection of 1×10^5^ 291OVA cells into either wild-type (Figure 1B) or GFP-transgenic mice (Figure 1C) led to a significant reduction and/or delay of lymphoma growth and strongly increased the survival of recipient mice compared to mice inoculated with (untransduced) 291PC cells. 15 of 20 wild-type (75%) and 17 of 25 of GFP-transgenic mice (68%) rejected the tumor within the 100 day observation period (Figure 1B: 291OVA vs. 291PC, p = 0.001 in wild-type recipients; Figure 1C: p = 0.01 in GFP-tg recipients). Similarly to OVA, also GFP acted as rejection antigen in this experimental system. 9 out of 15 wild-type mice (60%) rejected 291GFP cells, whereas all except one out of 25 GFP-transgenic, i.e. GFP-tolerant mice succumbed to lymphomas within 100 days (96%) (Figure 1C). Thus, OVA mediated lymphoma rejection *in vivo* in GFP-transgenic mice and, presumably synergistically with GFP, in wild-type mice. Animals inoculated with OVA-expressing lymphoma cells mounted a strong CD8+ T cell response specific for the immunodominant OVA-derived peptide SIINFEKL. CD90-selected splenic T cells from animals that rejected OVA-expressing lymphomas for over 50 days responded with IFN-γ secretion when restimulated either with peritoneal macrophages loaded with SIINFEKL peptide or with OVA-expressing lymphoma cells (Figure 2A). Notably, T cell responses were detectable at the earliest on day 7 after lymphoma transfer in the peripheral blood. Within 21 days after inoculation of 291OVA cells, up to 11% SIINFEKL-specific CD8+ cells appeared in the peripheral blood (Figure 2B). This T cell response was accompanied by massive infiltration of OVA-expressing lymphomas with CD3+ cells (Figure 2C).

Rejection of OVA expressing lymphomas in wild-type mice was confirmed in a second independent model system. λ-hu-MYC mice were crossbred with OVA-transgenic mice [23] and a cell line was established from a spontaneously developing OVA-transgenic λ-hu-MYC lymphoma (line 83OVA). Injection of 1×10^5^ 83OVA-transgenic lymphoma cells into wild-type mice resulted in 100% rejection of the inoculated cells (Figure S1), whereas in OVA-transgenic (i.e. OVA-tolerant) recipients seven of nine mice (78%) succumbed to locally as well as systemically growing lymphomas (p = 0.01).

### Antigen loss and increased MHC expression in lymphomas in mice receiving 291OVA cells

**Figure 3:**
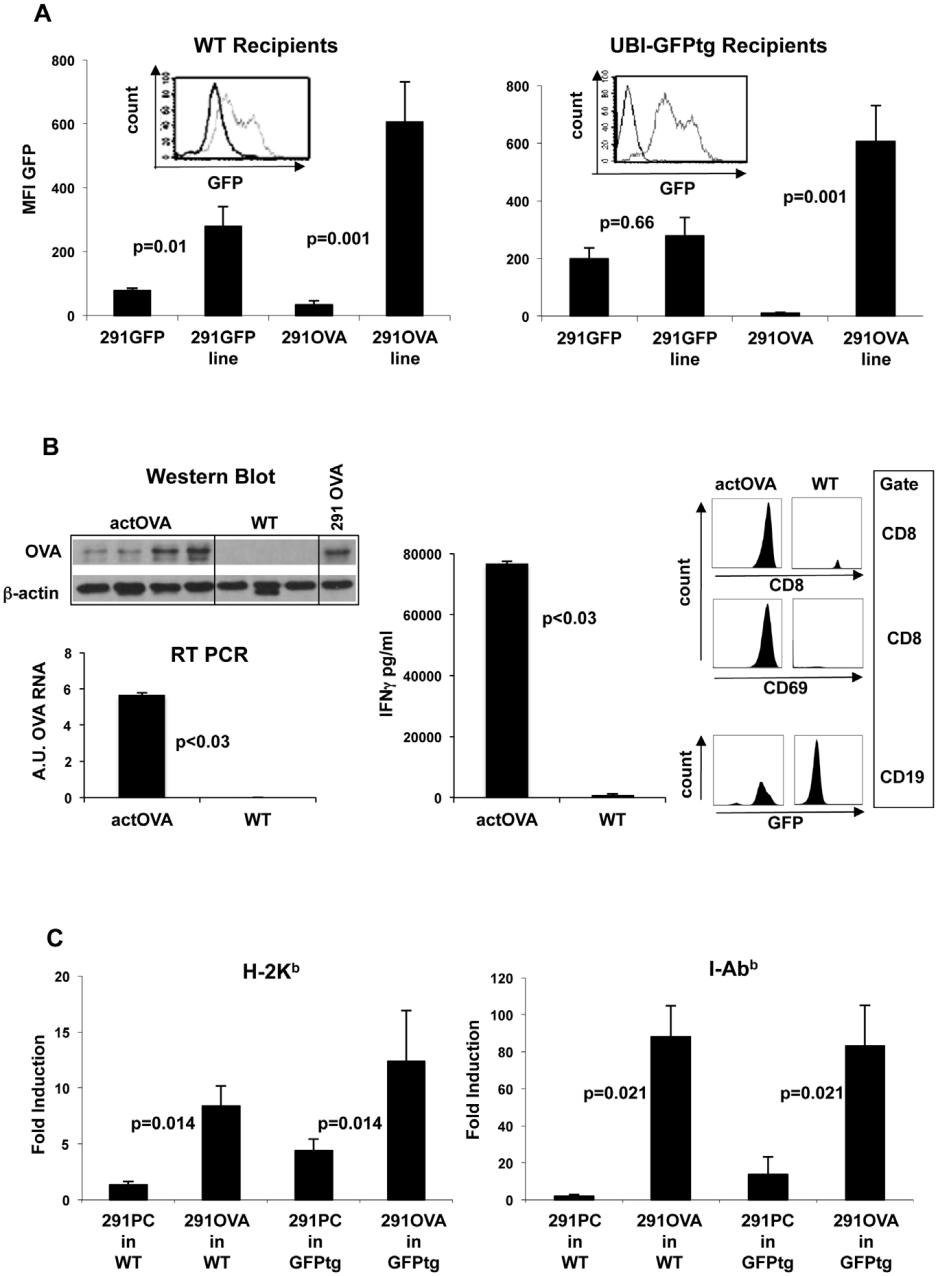
Transfer of OVA-expressing tumor cells into wild-type mice results in loss of antigen and induction of MHC on lymphoma cells. (A) Systemically growing lymphomas isolated from the spleen after s.c. inoculation of 291GFP or 291OVA cells were analyzed for GFP expression as a marker for antigen expression in comparison to the inoculated cell lines. Introduction of either GFP alone (291GFP) or OVA-IRES-GFP (291OVA) into lymphoma cells resulted in loss of or strong decrease in GFP expression in outgrowing lymphomas after transfer into wild-type mice (left panel, n = 6). Outgrowing lymphomas in GFP-transgenic recipients (tolerant for GFP) likewise lost GFP expression after inoculation of 291OVA cells, but retained the antigen when 291GFP cells had been inoculated (right panel). Flow cytometric histogram inlets show representative examples. Grey lines represent GFP expression at the time of injection, black lines after harvest of lymphomas from spleen. (B) Left: western Blot and RT-PCR analysis (arbitrary units, A.U.) of lymphomas harvested after s.c. inoculation of 291 OVA cells from either OVA tolerant (actOVA) or wild type (WT) recipients. In contrast to WT recipients OVA tolerant actOVA animals did not select for antigen (OVA) loss variants. Middle: Explanted lymphomas were cocultured with unprimed OT-I cells (1∶1 ratio). OVA positive lymphomas from actOVA recipients activated OT-I T-cells to secrete large amounts of IFN-γ (ELISA of supernatant, n = 4, Mann Whitney test) whereas OVA negatively selected lymphomas form wild type recipients did not stimulate OT-I cells. Right: Coculture of OT-I T-cells with lymphoma cells dervived from actOVA or wild type recipients resulted in induction of the T-cell activation marker CD69 expansion of CD8+ (OT-I) cells and reduction of the number of GFP expressing cells CD19+ lymphoma cells. Representative analysis from lymphoma cells harvested from individual mice. (C) Inoculation of 291OVA cells, but not of 291PC cells, into wild-type (n = 5) and GFP-transgenic mice (n = 5) significantly induced MHC class I (left panel) and class II (right panel) in outgrowing lymphoma cells. The strong increase in MHC class I and II expression in outgrowing lymphomas depended on the presence of OVA in the lymphoma inoculum (Mann Whitney test). Fold induction was calculated by comparing MHC expression on freshly explanted lymphoma cells with the corresponding cell line on the same day.

25% of wild-type mice developed lymphomas after inoculation of 291OVA cells (left panel of Figure 1B). Lymphomas growing systemically in the spleen as well as locally growing lymphomas were analyzed for GFP expression by FACS as surrogate marker for OVA expression. Lymphomas arising in wild-type mice after inoculation of 291OVA cells invariably displayed decreased GFP expression not only at the site of the primary lesion but also systemically (as shown for splenic lymphoma in Figure 3A). Likewise, GFP expression was strongly reduced in 291GFP lymphomas developing in wild-type mice (outgrowing lymphoma vs. injected cell line p = 0.01). Of note, in GFP-transgenic recipient mice, GFP expression was lost when 291OVA cells, but not when 291GFP cells were injected. Thus, outgrowing lymphomas escaped rejection by either downregulation or loss of antigen expression. Similar results were obtained when 291OVA cells were analyzed *in vitro* after outgrowth in either OVA tolerant actOVA transgenic recipients or in wild-type animals. As shown in figure 3B left panel, lymphomas outgrowing in wild type recipients expressed neither OVA on a protein level (western blot) nor on RNA level (RT-PCR) in contrast to lymphomas outgrowing in actOVA animals. Consequently, OVA negatively selected lymphoma cells that escaped *in vivo* T cell pressure, failed to stimulate OT-I cells *in vitro* to secrete IFN-γ (Figure 3B, middle panel). In line with these results, coculture of OT-I T cells with lymphoma cells derived from actOVA recipients resulted in induction of the T cell activation marker CD69 and expansion of CD8+ (OT-1) cells. (Figure 3B, upper right panel). In contrast, antigen negatively selected lymphomas from wild type recipients (WT), failed to induce CD69 and expansion of CD8+ (OT-I) T-cells, and continued to proliferate in coculture (Figure 3B lower right panel). In contrast, the number of GFP expressing lymphoma cells derived from actOVA recipients was reduced after 48 h of coculture with OT-1 T cells.

Lymphomas growing out after inoculation of 291OVA cells into wild-type or GFP-transgenic mice expressed MHC class I and II more strongly than lymphomas induced by inoculation of 291PC cells (Figure 3C).

### Host IFN-γ and host IFN-γ-responsiveness are required for rejection

**Figure 4:**
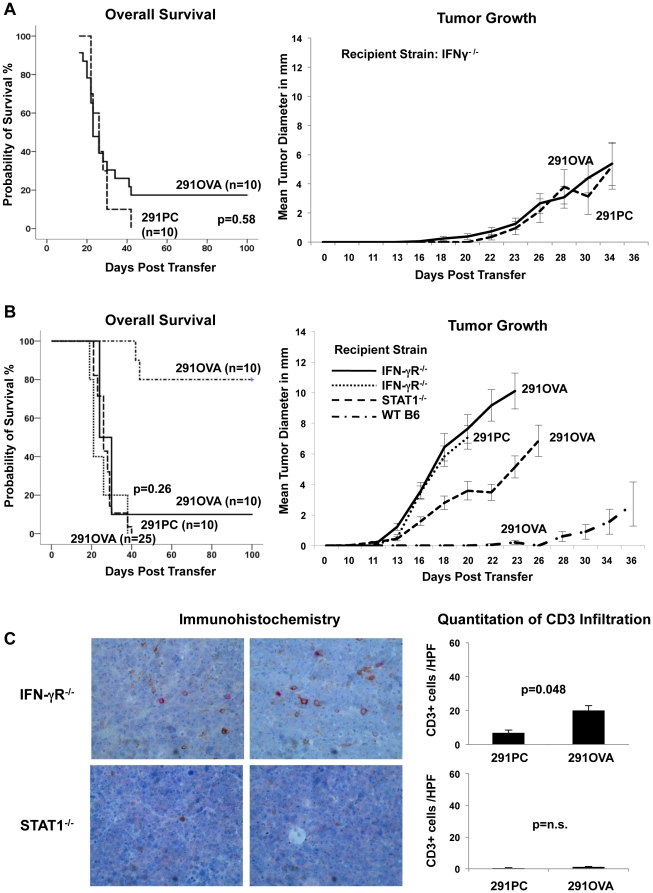
Host IFN-γ and host IFN-γ signaling are required for rejection of 291OVA cells. 1×10^5^ 291 parental cells (291PC) and retrovirally transduced 291OVA cells were injected s.c. into IFN-γ-deficient (A) and into IFN-γ-receptor- and STAT1-deficient recipient mice (B). Survival (left panels) and corresponding cumulative tumor growth (right panels) were monitored over 100 days. The data are compiled from two independent experiments. (C) Lymphomas developing in IFN-γ-receptor- and STAT1-deficient mice after inoculation of 1×10^5^ 291 parental or 291OVA cells were analyzed by immunohistochemistry for infiltration of CD3-positive cells (peroxidase brown staining) and perforin expressing cells (alkaline phosphatase staining) (left panel) in the same fashion as shown in Figure 2C. 10 animals per group were analyzed and Mann Whitney test was used for comparison.

In this model lymphoma rejection depends on foreign antigen expression. The marked increase in SIINFEKL-pentamer-positive T cells in mice rejecting the tumors and the elimination of antigen expression in 291OVA cells cocultured with *in vivo*-primed OT-I cells *in vitro* point to a T cell-mediated mechanism of rejection. Given the importance of IFN-γ as effector molecule of T cells, we asked whether rejection depends on IFN-γ. 291OVA cells were injected into IFN-γ-deficient recipient animals, and lymphoma growth was monitored over time. As shown in Figure 4A, IFN-γ deficiency in the recipient led to almost complete loss of protection, and 80% of the recipient mice died from outgrowing lymphomas. The kinetics of tumor onset and speed of tumor growth were not markedly different after injection of 291OVA cells and untransduced 291PC cells (Figure 4A, right panel), with the difference that 20% of the IFN-γ-deficient animals receiving 291OVA cells remained tumor-free for at least 100 days. To ask whether IFN-γ acts on the side of the recipient, 291OVA and 291PC lymphoma cells were injected into IFN-γ-receptor- and STAT1-deficient mice lacking essential components of the interferon signaling system. Figure 4B shows that loss of responsiveness to both types of interferons (STAT1^−/−^ recipients) resulted in 100% mortality after inoculation of 291OVA cells. No infiltration of T cells was observed in STAT1^−/−^ recipients that had received 291OVA cells suggesting that recruitment of antigen-specific T cells to the tumor site is severely impaired in STAT1^−/−^ mice (bottom panel, Figure 4C). In recipient mice lacking IFN-γ receptor (IFN-γR^−/−^) onset of lymphoma growth after inoculation occurred at the same time as in STAT1^−/−^ recipients and lymphomas appeared to grow out slightly faster in IFN-γR^−/−^ recipients. From day 18 on mean tumor diameters were significantly different in IFN-γR^−/−^ and STAT1^−/−^ recipients (p = 0.046–0.013, Mann Whitney test), but this difference did not result in differences in survival. In IFN-γR^−/−^ recipients absence of antigen (291PC) did not result in faster lymphoma growth. T cell infiltration was observed in IFN-γR^−/−^ recipients after inoculation of 291OVA cells in contrast to STAT1^−/−^ recipients (Figure 4C), although to a lesser extent than in wild-type recipients (shown Figure 2C). These results show that both IFN-γ and IFN-γ-signaling are both required in the host to achieve antigen-dependent lymphoma rejection.

**Figure 5:**
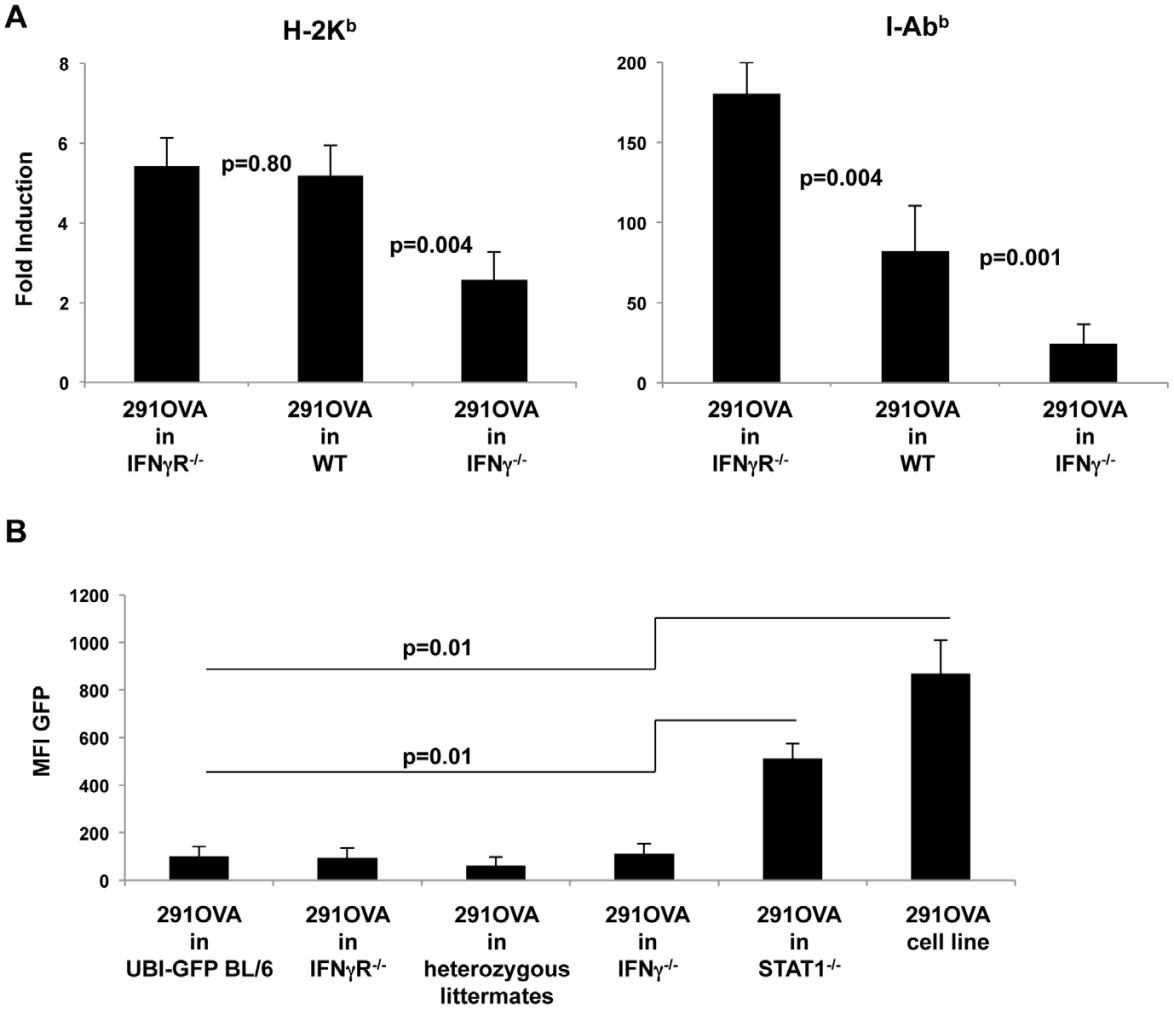
Loss of antigen expression in IFN-γ or IFN-γ-receptor-deficient, but not in STAT1-deficient animals. (A) Upregulation of MHC class I and II expression in lymphomas arising in wild-type and in IFN-γ-receptor-deficient mice after inoculation of 291OVA cells. Values are given as a mean (± standard error of the mean) of fold-induction based on values obtained from 291OVA cells in culture (n = 13–22 animals per group). (B) Antigen expression was maintained in outgrowing lymphomas after inoculation of 291OVA cells in STAT1^−/−^ mice, but was invariably lost after inoculation of 291OVA cells into wild-type, IFN-γ^−/−^ and IFN-γ-receptor^−/−^ mice and their heterozyogous littermates (compiled IFN-γ^+/−^ and IFN-γR^+/−^) (n = 8–15, p = 0.01), comparison of GFP expression in the 291OVA cell line and in STAT1^−/−^ recipients with that in all other recipients. Mean fluorescence intensity of GFP in outgrowing lymphomas as a surrogate marker for OVA expression.

We next asked whether the presence of IFN-γ or IFN-γR^−/−^ in recipient mice affects MHC expression in tumors arising after inoculation of 291OVA cells. The degree of MHC class I and II induction was significantly reduced in lymphomas arising in IFN-γ^−/−^ recipients compared to wild-type controls (Figure 5A). However, a similar degree of MHC class I induction and even a higher degree of MHC class II induction was observed in IFN-γR^−/−^ recipient compared to wild-type mice.

Having observed that (i) 291OVA cells were not or only poorly rejected in IFN-γ^−/−^, IFN-γR^−/−^, and STAT1^−/−^ recipient mice (Figure 4), and (ii) that IFN-γ expression in the host was required to upregulate MHC expression in tumor cells (Figure 5A), we asked whether antigen expression was maintained or lost in IFN-γ^−/−^, IFN-γR^−/−^, and STAT1^−/−^ recipient mice after inoculation of 291OVA cells. Lymphomas arising in STAT1^−/−^ mice invariably expressed GFP consistent with the lack of T cell immigration to the tumor site (Figure 3C). However, all excised lymphomas from IFN-γ^−/−^ and IFN-γR^−/−^ recipients were negative for GFP, indicating that OVA was not expressed in the lymphomas (Figure 5B). This indicated that in IFN-γ^−/−^ and IFN-γR^−/−^ recipients, the T cell response was sufficient to eliminate OVA-expressing lymphoma cells. However, the elimination of the OVA-expressing cell population within the tumor neither decreased lymphoma incidence nor influenced the kinetics of lymphoma outgrowth to a significant extent and did not improve overall survival (Figure 4).

### IFN-γ-responsiveness of lymphoma cells is not required for antigen-dependent rejection

**Figure 6:**
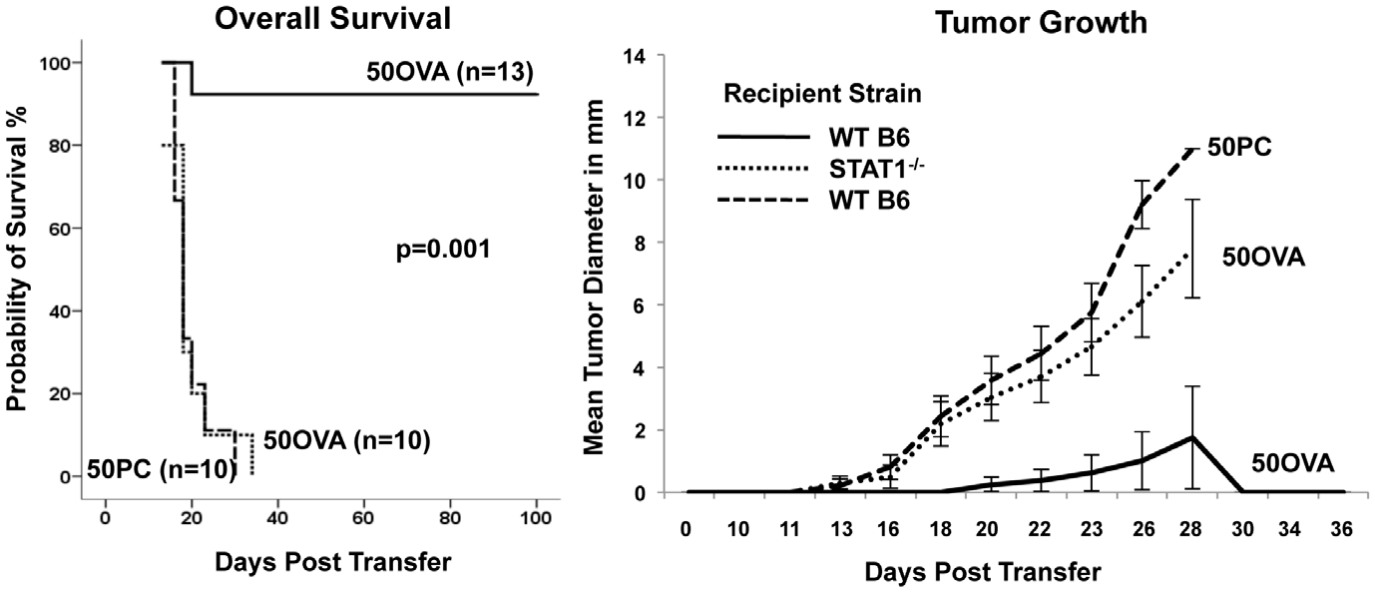
IFN-γ responsiveness of lymphoma cells is not required for antigen-dependent lymphoma rejection. IFN-γ-receptor-deficient λ-huMYC lymphoma cells, either retrovirally transduced (50OVA) (solid line) or untransduced (50PC) (dashed line), were injected s.c. into wild-type mice. STAT1-deficient mice inoculated with retrovirally transduced 50OVA cells served as positive control (dotted line). Mice were monitored for survival (left panel) and cumulative tumor growth (right panel). The data are combined from 2 independent experiments.

The requirement of host IFN-γ for lymphoma rejection can be explained by two non-mutually exclusive mechanisms: a direct action of IFN-γ on the tumor cells [24] or an effect of IFN-γ on the host [18]. To address whether IFN-γ-responsiveness of the tumor is required for lymphoma rejection, λ-hu-MYC mice were crossed onto syngeneic IFN-γ-receptor-deficient or STAT1-deficient mice. Cell lines were established from spontaneously arising lymphomas. One cell line established from an IFN-γR^−/−^ lymphoma (50PC) was retrovirally transduced with OVA-IRES-GFP. The parental line and the retrovirally transduced line were inoculated into wild-type and into STAT1-deficient mice as a positive control. The parental untransduced line displayed tumorigenic potential with 100% mortality after inoculation into wild-type mice (Figure 6). The OVA-IRES-GFP-transduced line (50OVA) was similarly tumorigenic in STAT1^−/−^ mice. In contrast, IFN-γ-receptor-deficient OVA-IRES-GFP-transduced cells were readily rejected in wild-type mice, and in only one out of 13 cases a lymphoma grew out resulting in 92.7% overall survival (Figure 6). The finding that OVA-expressing lymphomas lacking the IFN-γ receptor were readily rejected indicated that IFN-γ responsiveness of the tumor cells had little if any impact on tumor rejection in this model.

Similar results were obtained with STAT1-deficient lymphoma cells (line 9PC, 9OVA, table 1). A STAT1-deficient cell line (9PC) established from a spontaneous lymphoma was retrovirally transduced with OVA-IRES-GFP (9OVA) and injected into either wild-type or STAT1-deficient recipients. In accordance with the data shown above for OVA-expressing lymphomas lacking the IFN-γ receptor, approximately 85% of wild-type recipients but none of the STAT1-deficient recipient mice rejected STAT1-deficient lymphoma cells expressing OVA.

**Table 1:** Cell lines used for this study.

Cell line	Genotype	Lymphoma-specific antigen	Rejection in wild type B6
291PC	λ-hu-MYC tg	huMYC	0%
291GFP	λ-hu-MYC tg	huMYC/GFP	60%
291OVA	λ-hu-MYC tg	huMYC/GFP/OVA	75%
83OVA	λ-hu-MYC; tg Act-OVA tg	OVA	100%
9PC	STAT1^−/−^ λ-hu-MYC tg	huMYC	30%
9OVA	STAT1^−/−^; λ-hu-MYC tg	huMYC/GFP/OVA	82%
50PC	IFN-γ-R^−/−^; λ -hu-MYC tg	huMYC	0%
50OVA	IFN-γ-R^−/−^; λ -hu-MYC tg	huMYC/GFP/OVA	93%

Overview of genotype potential foreign antigens and rejection of lymphoma cell lines used in this study. Numbers are given as % of rejection after s.c. injection of 1×10^5^ lymphoma cells from cell lines indicated into recipient wild type C57BL/6 mice.

### Defective cross-presentation in host tissue results in impaired T cell response and faster lymphoma growth

**Figure 7:**
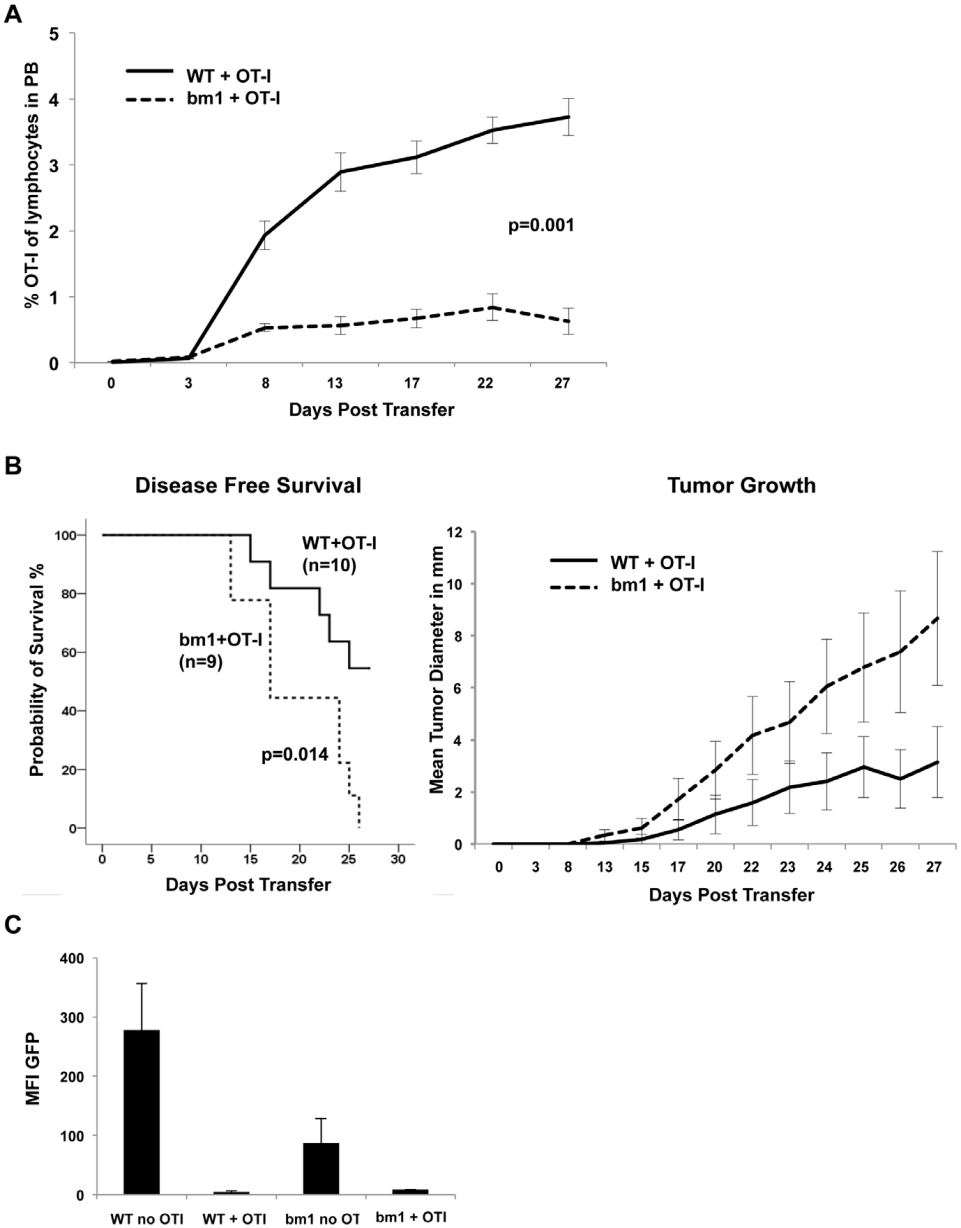
Defective cross-presentation in cells of the recipient impairs T cell response and enhances lymphoma growth. Recipient animals were T cell depleted as described in Materials and Methods and T cell depletion was continued for 28 days. Wild-type (WT) or bm1 mutant recipient mice received 1×10^5^ 291OVA lymphoma cells s.c. together with 1×10^6^ *in vivo* primed OT-I cells. (A) Development of an OVA-specific T cell response is impaired in bm1 recipients. Peripheral blood of animals inoculated with 1×10^5^ 291OVA and 1×10^6^ OT-I cells were analyzed by flow cytometry for the presence of CD90.1-positive cells (OT-I) on the days indicated. Numbers of OT-1 T cells are expressed as percentage of lymphocytes in the peripheral blood. In wild-type mice (solid line, n = 10) adoptively transferred OT-I cells expand more readily (Mann Whitney test) than in bm1 recipients (dashed line, n = 9, p = 0.001 for day 8–27). (B) Disease-free survival and cumulative tumor growth after lymphoma transfer: bm1 recipient mice (dashed line) developed tumors significantly faster and cumulative lymphoma growth was enhanced (right panel). (C) Mean fluorescence of GFP in lymphomas arising in wild-type mice or bm1 mice in the presence and absence of OT-I cells. T cell depletion in the absence of OT-I cells (WT no OT-I, n = 9) resulted in preservation of antigen expression, while adoptive transfer of OT-I cells (WT+OT-I, n = 5) led to selection of antigen-negative lymphoma cells.

To address the role of the stroma for lymphoma rejection, we made use of C57BL/6^H2K bm1^ mice that harbor a mutation in the H-2K class I locus preventing the presentation of SIINFEKL peptide by the stroma [25], [26]. The bm1 mutation is recognized as an alloantigen [27]. Therefore, we T cell-depleted recipient animals by injection of the anti-CD90.2 antibody 30H12 one day before lymphoma transfer and biweekly thereafter. This antibody treatment caused a 2-log reduction in peripheral blood T cell numbers. This reduction was maintained over 28 days by repeated antibody treatment. Either wild-type or bm1 mutants, were inoculated s.c. with 1×10^5^ 291OVA cells on day 0 and received 1×10^6^ primed CD90.1 positive OT-I cells on day 1. Anti-CD90.2 antibody treatment depleted endogenous CD90.2-positive T cells but not CD90.1-positive OT-I cells. Animals harboring the bm1 mutation had significantly reduced T cell expansion within the peripheral blood compared to wild-type recipients (Figure 7A). Within the observation period of 28 days, all bm1 mice but only 50% of wild-type animals succumbed to lymphomas (Figure 7B, left panel) and cumulative lymphoma growth was significantly more pronounced in bm1 than in wild-type mice (right panel). Outgrowing lymphomas invariably were antigen-negative (as revealed by GFP as a surrogate marker for antigen expression), regardless whether lymphomas arose in bm1 mice or wild-type mice (Figure 7C), indicating that direct antigen-dependent killing had occurred in wild-type as well as in bm1 animals.

These findings indicate (i) that direct antigen-dependent T cell killing is responsible for the eradication of antigen-expressing lymphoma cells, and (ii) that antigen cross-presentation by non-tumor cells is required to prevent lymphoma growth of antigen-loss variants.

## Discussion

### OVA and GFP serve as rejection antigens in the λ-hu-MYC model

The advent of cloning TCRs with high avidity and their transduction into T cells opens new avenues for cellular immunotherapy. The therapeutic potential of these novel strategies, however, is closely linked to the risk of uncontrollable auto-immunity similar to life-threatening GvHD after allogeneic SCT. We reason that B cell leukemias and lymphomas will become an important paradigm for the evaluation of this treatment option, because auto-immunity, if restricted to the B cell compartment, is compatible with life. We set up a mouse model that explores the power as well as potential pitfalls of adoptive T cell therapy.

Since high-avidity TCRs for B cell differentiation antigens are not yet available, we have introduced chicken OVA into λ-hu-MYC lymphoma cells as a foreign antigen for which MHC class I- as well as class II-restricted TCR-transgenic mice and epitope-specific MHC class I-pentamers are available. We utilized two types of lymphomas, λ-hu-MYC lymphomas into which OVA-IRES-GFP had been introduced by retroviral transduction as well as lymphomas arising spontaneously in β-Act-OVA/λ-hu-MYC double transgenice mice. Besides OVA, human c-MYC (in the double transgenic model) or human c-MYC plus GFP (in the retrovirally transduced lymphoma model) are expressed as foreign antigens whose potential as tumor rejection antigens have not been explored. The latter is particularly important as GFP is frequently introduced into tumors without accounting for its immunogenic potential [28]. When OVA, GFP, or OVA plus GFP were expressed in these cells, tumors were rejected except for a few cases in which tumor outgrowth resumed after a considerable delay. OVA- and/or GFP-expressing lymphomas were rejected at a dose of 1×10^5^ cells without prior immunization, but human c-MYC-expressing lymphomas were not rejected.

The increase in SIINFEKL-MHC-class I-pentamer-specific T cells from 0.8% up to 11% and the strong IFN-γ production of OT-I T cells upon stimulation with antigen-expressing tumor cells indicated that rejection was mediated by OVA-specific (and presumably also by GFP-specific) T cells. In about 25% of the cases, lymphomas grew out with delayed kinetics, were GFP-negative and had upregulated MHC class I and class II expression, representing cells that had either been selected for loss of antigen expression *in vivo* [29], or more likely, for cells that had not been retrovirally transduced. Since the purity of FACS-sorted lymphoma cells ranged between 95 and 99%, approximately 1–5×10^3^ untransduced GFP-negative lymphoma cells were injected.

### Interferon-γ signaling in the host, and not in the tumor, is required for efficient rejection

To address whether antigen-dependent lymphoma rejection depends on IFN-γ signaling in the host or in the tumor cells, OVA-GFP-expressing wild-type lymphoma cells were inoculated into either IFN-γ^−/−^ or IFN-γR^−/−^ mice, and on the other hand, antigen-expressing lymphoma cells deficient for either the IFN-γ receptor or STAT1 were inoculated into wild-type mice. Contrary to wild-type mice, neither IFN-γ^−/−^ nor IFN-γR^−/−^ mice prevented outgrowth of OVA-GFP-transduced lymphoma cells resulting in poor overall survival. In contrast, the vast majority of wild-type mice inoculated with OVA-GFP-transduced lymphoma cells deficient for the IFN-γ receptor or STAT1 rejected the tumor cells and survived the observation period of 100 days indicating that IFN-γ signaling in the host but not in the tumor is required for rejection of OVA-GFP-transduced lymphomas.

### Interferon-γ signaling in the host is required to eliminate antigen-loss variants efficiently

When comparing overall survival, no significant differences between IFN-γ^−/−^, IFN-γR^−/−^, and STAT1^−/−^ mice inoculated with OVA-GFP-transduced lymphoma cells were observed. Moreover, transduction of lymphoma cells with an OVA-GFP-encoding retrovirus prior to inoculation did not influence overall survival significantly in all three mouse strains deficient in IFN-γ signaling. Notably, tumors arising in IFN-γ^−/−^ and IFN-γR^−/−^ mice were invariably GFP-negative, whereas all lymphomas arising in STAT1^−/−^ mice were GFP-positive. Apparently, T cells were not recruited to the site of antigen production in STAT1^−/−^ mice (Figure 4C), and thus foreign antigen expression was not a selective disadvantage in STAT1−/− recipient mice. In contrast, in IFN-γ^−/−^ and IFN-γ-R^−/−^ mice antigen-expressing tumors were apparently counterselected and antigen-negative cells grew out efficiently. Thus, antigen-positive lymphoma cells can be eliminated in an IFN-γ- and IFN-γR-deficient host in a similar manner to wild-type mice, but tumor outgrowth and overall survival depends greatly upon eradication of antigen-negative lymphoma cell variants.

### Antigen cross-presentation is required to eliminate antigen-loss variants of the tumor

Our observations are reminiscent of findings by Schüler and Blankenstein in a transplanted OVA-expressing B16 melanoma model [30] and of Hans Schreiber and collaborators obtained in a chemical carcinogen-induced murine sarcoma model [19]–[21]. The latter is a highly immunogenic tumor that is rejected in wild-type mice. It was converted into a tumor model for adoptive T cell therapy (i) by using OT-I mice as recipients that harbor a skewed T cell repertoire and cannot reject the tumor, and (ii) by expression of SIY and gp33 at high and low levels in the sarcoma cells which makes them vulnerable to transferred SIY- and gp33-specific T cells from TCR-transgenic mice [19]–[21]. They showed that antigen-specific T cells can kill antigen-loss variants in an IFN-γ- and TNF-α-dependent manner and can cure mice with an even larger tumor burden. Antigen-specific T cells eradicated tumor stroma cells that cross-present the tumor antigen from dying tumor cells, thus withdrawing the tumor-supportive microenvironment.

To see whether a similar mechanism holds true for B cell lymphomas, we transplanted antigen expressing λ-hu-MYC lymphoma cells into T cell-depleted bm1 mice and treated the mice with OT-I T cells. OT-I T cells can attack tumor cells, but cannot recognize cross-presented antigen on bm1 stroma cells. Indeed, antigen-negative lymphomas grew out within four weeks in all 291OVA-inoculated bm1 mice but only in 50% of wild-type mice supporting the notion that elimination of antigen-loss variants requires antigen cross-presentation by non-tumor cells, presumably by stromal cells.

Provided that the bm1 mutation would lead to NK cell activation, the reduced expansion of adoptively transferred OT-I cells might also be a result of NK-cell mediated resistance in bm1 recipients in the peripheral blood. The fact that almost all antigen positive lymphoma cells are eliminated strongly suggest that not insufficient T cell recognition of lymphoma cells and numbers of T cells but rather (similar to what has been observed for solid tumors) the elimination of stromal support by the destruction of cross presenting stromal cells is responsible for the differences shown in Figure 7B and 7C.

The similarities between a chemical carcinogen-induced murine sarcoma and our λ-hu-MYC lymphoma model into which OVA and GFP were introduced as foreign antigens are intriguing. The carcinogen-induced sarcoma is intrinsically highly immunogenic and rejected in wild-type mice even in the absence of SIY and gp33 (regressor phenotype). In contrast, the λ-hu-MYC lymphoma is a poorly immunogenic, highly malignant tumor that rapidly progresses in wild-type mice in the absence of OVA and/or GFP (progressor phenotype). This is to our knowledge the first report of T cell-mediated antigen-dependent bystander killing of tumor antigen-loss variants in mice with a normal T cell repertoire and a non-lymphopenic environment.

Stroma cells play an important role in sustaining survival and proliferation of tumor cells in chronic lymphocytic leukemia [31], multiple myeloma [32] and Hodgkin's disease [33]. In Hodgkin's disease the Reed-Sternberg tumor cells comprise only a minority of the tumor suggesting that surrounding non-malignant T cells play an auxiliary role. For the murine Eμ-myc model, human NHLs and multiple myeloma, hedgehog cell signaling by stroma cells plays an important tumor-supportive role [34]. Gene expression signatures of stroma cells isolated from diffuse large B cell lymphomas (DLBCLs) *ex vivo*, have been correlated with favorable or adverse prognosis [35]. Moreover, in the Eμ-myc mouse lymphoma model, apoptotic tumor cells cause secretion of TGF-β by macrophages in the tumor stroma thus inducing cellular senescence [36]. Thus, stroma cells not only contribute to tumor progression but can also limit tumorigenesis. In the sarcoma model discussed above, both non-bone marrow-derived cells as well as bone marrow-derived myeloid cells are necessary to control antigen-loss variants by antigen-specific T cells [37]. Immature myeloid stroma cells can act immunosuppressively in many malignancies in mice and man [38], [39].

Although the nature of stroma cells in our λ-hu-MYC lymphoma model requires further investigation, our data support an auxiliary role of stroma cells in a murine high-grade NHL model and stress the importance of targeting the stroma during immunotherapy for non-solid tumors as well. In our model we do not know which type of cells within the stroma of the lymphomas contribute to the effects seen in this study and further experiments using hematopoietic chimeras are necessary to clarify this important question. Chemotherapeutic agents and irradiation induce tumor cell death and thereby increase cross-presentation of tumor antigens by stroma cells [20]. In addition, chemotherapy induces lymphopenia which is favorable for the expansion of adoptively transferred T cells. Thus, adoptive T cell and conventional tumor therapy may synergize in tumor eradication by preventing outgrowth of antigen-loss variants.

## Materials and Methods

### Mice

All mice were of C57BL/6 background (henceforth referred to as wild-type). λ-hu-MYC male mice were bred to wild-type females. Offspring were genotyped for the transcript by PCR using primers as published [22].

For some experiments λ-hu-MYC mice were backcrossed to IFN-γ^−/−^ (129S7-*Ifng^tm1Ts^*/J), IFN-γR^−/−^ (.129S7-*Ifngr1^tm1Agt^*/J) or STAT1^−/−^ [40] kindly provided by David E. Levy (New York) through Matthias Müller (Vienna). F1 generations were backcrossed into the knock out strains giving rise to lymphomas of IFN-γ^−/−^, IFN-γR^−/−^ or STAT1^−/−^ genotype. OT-I mice (-Tg(TcraTcrb)1100 Mjb/J), mice transgenic for GFP (ubiquitin promoter-driven GFP expression) [41] and mice transgenic for OVA (beta actin promoter-driven OVA expression) (−Tg(Actb-OVA)916Jen/J) [23] were purchased from Jackson Laboratories, Bar Harbor, Maine, USA. Wild-type mice were purchased from Charles River Laboratories (Germany). Wild-type, RAG1^−/−^, IFN-γ^−/−^, IFN-γR^−/−^, STAT1^−/−^ and C57BL/6^H2kbm1^ recipient mice were bred in our facility under specific pathogen free conditions (SPF). Only sex-matched recipients of similar age (10–14 weeks) were used for transfer experiments.

All experiments were performed according to guidelines and approval of the local animal protection committee (application: 55.2-1-54-2531-8-04 and 209.1/211-2531-8/04, Government of Bavaria, Munich, Germany).

### Immunization of OT-I mice

T cells from OT-I mice were primed by subcutaneous immunization with 50 µg chicken OVA (Sigma, Germany) in combination with incomplete Freund's adjuvant. CD8+ T cells were isolated with anti-CD8 magnetic beads (Miltenyi, Germany) at day 7 after immunization and used for assays or i.v. transfer into recipient mice as indicated.

### Cell lines, cell culture, and retroviral transduction

Developing lymphomas were explanted, resuspended, and plated onto irradiated MRC5 feeder cells in Iscoves IMDM Media (GibcoBRL, Germany) supplemented with 20% FCS, 2 mM glutamine, 100 U/ml Penicillin, 100 µg/ml Streptomycin, 1 mM sodium pyruvate, 20 nM bathocuproine disulfonate and 50 µM α-thioglycerol [42]. 291 cells were chosen as representative cells that display feeder-independent growth in culture, express high amounts of human c-MYC protein and low amounts of MHC class I, and virtually no class II in culture.

Parental cells (PC) from established cell lines (291PC, 50PC (IFN-γR^−/−^), 9PC (STAT1^−/−^) were transduced with retroviral MSCV-based vectors [43] expressing OVA-IRES-GFP or IRES-GFP alone giving rise to GFP or OVA cell lines (see below). Using the Phoenix packaging cell line, retroviral supernatants were generated as described [44]. High titer viral supernatant was passed through a 0.45 µm filter and supplemented with 4 µg/ml polybrene (Sigma, Germany). Three ml of the viral supernatant were used to resuspend pellets of 5 million lymphoma cells. The infection procedure was repeated twice at 12 hours intervals. After 48–72 h GFP expression was determined by flow cytometry and 10–15% of virus-transduced lymphoma cells were positive for GFP. Retrovirally transduced cells were sorted for high GFP expression by FACS and 95–98% purity of GFP expressing cells was obtained. As GFP is the second gene expressed from a bicistronic transcription cassette, expression of GFP can be reliably taken as a surrogate for OVA expression [45], [46].

The cell line 83OVA was established from double transgenic β-actin-OVA, λ-hu-MYC mice. These mice were generated by crossing β-actin-OVA-transgenic mice [23] (Jackson Laboratories, Bar Harbor, Maine) to λ-hu-MYC mice [22]. An overview of cell lines used in this study is given in table 1.

Incubation with 100 IU murine IFN-γ for 24 h induced MHC class I and II expression in 291OVA cells, but not in IFN-γR-deficient 50OVA and in STAT1-deficient 9OVA cells (Figure S2).

### Tumor transfer and monitoring

For lymphoma transfer experiments, cells from cultured lymphomas (line 291PC, GFP or OVA, line 9PC or OVA and line 50PC or OVA) were freshly split 48 h prior to transfer. Cells were washed in cold PBS and injected s.c. in the upper flank of recipient mice. Lymphoma growth was measured at the site of injection at least 3 times per week as indicated in the figures using a sliding caliper. Tumor size is expressed as average of cumulative data (± standard error of the mean) from all mice injected with lymphoma cells.

### T cell depletion of recipient mice

In some experiments we depleted lymphoma recipient mice of T cells by intraperitoneal injection of 1 mg 30H12 anti CD90.2 antibody 1 day before lymphoma transfer and biweekly thereafter. Depletion was monitored by FACS of peripheral blood using anti-CD4 or anti-CD8 antibody.

### Flow cytometric analysis

Cells were washed in freshly prepared, ice cold PBS/5% FCS and incubated using 10% supernatant from clone 2.4G2, to block Fcγ receptors for 30 min on ice. FITC- or PE-labeled monoclonal antibodies were applied and incubated for 30 min on ice. Cells were washed twice in cold PBS/FCS and subsequently analyzed (FACSCalibur, Becton Dickinson, Heidelberg, Germany). Pentamers (ProImmune, Oxford, UK) were used according to the manufacturer's protocol.

### Histology and immunohistochemistry

Mice were killed by CO_2_ asphyxiation. Parts of the lymphomas were fixed in 10% formalin for 12 h. For immunostaining of paraffin embedded material, 2–3 µm sections were cut, deparaffinized and subjected to a heat-induced epitope retrieval step before incubation with antibodies. Sections were immersed in sodium citrate buffer solutions at pH 6.0 and heated in a high-pressure cooker. The slides were washed in Tris-buffered saline (pH 7.4) and incubated with primary antibodies against perforin (P1-8, 1∶100) and CD3 (1∶10). For detection, biotinylated rabbit anti-rat (Dako) or donkey anti-rabbit (Dianova, Hamburg, Germany) secondary antibodies were used, followed by the streptavidin-AP kit or the Envision-PO kit (Dako). Alkaline phosphatase was revealed by Fast Red as chromogen and peroxidase was developed with a highly sensitive diaminobenzidine (DAB) chromogenic substrate for approximately 10 min.

### Primary antibodies used

For flow cytometry the following antibodies were used: CD19 (clone 1D3), H-2Kb (clone AF6-88.5), IAb (clone 25-9-17), CD80 (clone 16-10A1), CD86 (clone GL1), CD54 (clone 3E2), CD4 (clone RM4-5), CD8 (clone 53-6.7), CD3 (clone 145-2C11), TCR Vα2 (clone B20.1), TCR Vβ5.1 (clone MR9-4), all from Becton Dickinson, (Heidelberg, Germany).

For immunohistochemistry the following antibodies were used: CD3 (Dako, Glostrup, Denmark), perforin (clone P1-8, Hoelzel Diagnostika, Cologne, Germany).

### Light Cycler PCR

Total RNA was isolated from cell lymphoma samples according to the manufacturer's instructions using a Qiagen RNeasy Plus Mini kit (Qiagen, Germany). After DNAse treatment (New England Biolabs) cDNA was prepared by reverse transcription with Random Decamers (Applied Biosystems, Germany) as suggested by the manufacturer. cDNA was analysed by quantitative real time PCR (ABI step one) using the following primers (selected by Pearlprimer software; Metabion, Germany): 18S RNA forward 5-CGCCGCTAGAGGTGAAATTC-3, reverse 5-CGAACCTCCGACTTTCGTTCT-3. OVA-RNA forward 5-GGAGCTTCCATTTGCCAGTGG-3, reverse 5-AGAGACGCTTGCAGCATCCAC-3. C_t_ values were quantified using appropriate software (Applied Biosystems, Germany) and expressed in arbitratry units (A.U.).

## Supporting Information

Figure S1 **Act-OVA transgenic animals fail to reject 291OVA cells.** λ-hu-MYC transgenic mice were crossbred with Act-OVA-transgenic animals and cell lines established from spontaneously arising lymphomas in double transgenic mice. 1×10^5^ cells of the cell line 83OVA were injected s.c. into either wild-type or Act-OVA- transgenic recipients. Left panel: wild-type animals rejected OVA-expressing lymphoma cells (dotted line), whereas OVA-tolerant Act-OVA-transgenic recipients succumbed to rapidly growing tumors (solid line). Right panel: corresponding growth curve of lymphomas representing the cumulative tumor diameter of all lymphomas at the site of injection.
(TIF)

Figure S2 **Induction of MHC class I and II by IFN-γ is dependent on STAT1- and IFN-γ receptor-signaling.** 291OVA (wild-type), 9OVA (STAT1^−/−^), and 50OVA (IFN-γ-R^−/−^) were exposed to 100 U IFN-γ for 24 hours and MHC class I expression was assessed by flow cytometric analysis. STAT1-deficient and IFN-γ receptor-deficient lymphoma cells do not upregulate MHC class I upon IFN-γ treatment.
(TIF)
