# Supplementary figures and images for: Stromal Interferon-γ Signaling and Cross-Presentation Are Required to Eliminate Antigen-Loss Variants of B Cell Lymphomas in Mice
Source: PLoS One. 2012 Mar 30;7(3):e34552. doi: 10.1371/journal.pone.0034552 (PMC3316708; doi:10.1371/journal.pone.0034552)

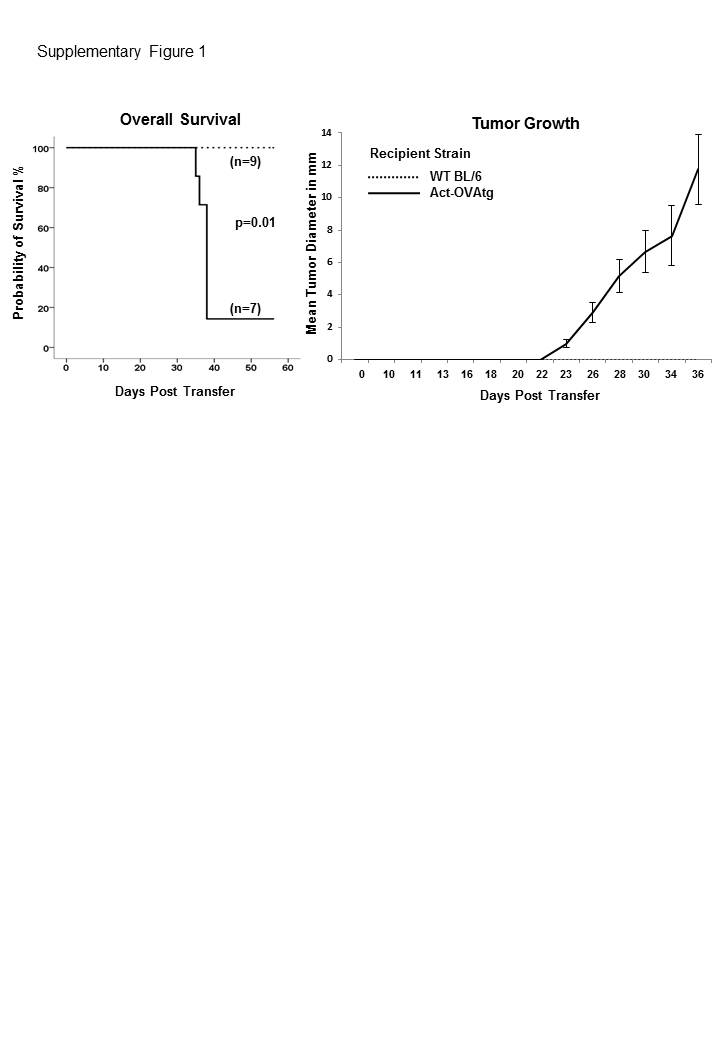

Supplement: Figure S1 — Act-OVA transgenic animals fail to reject 291OVA cells. λ-hu-MYC transgenic mice were crossbred with Act-OVA-transgenic animals and cell lines established from spontaneously arising lymphomas in double transgenic mice. 1×10^5^ cells of the cell line 83OVA were injected s.c. into either wild-type or Act-OVA- transgenic recipients. Left panel: wild-type animals rejected OVA-expressing lymphoma cells (dotted line), whereas OVA-tolerant Act-OVA-transgenic recipients succumbed to rapidly growing tumors (solid line). Right panel: corresponding growth curve of lymphomas representing the cumulative tumor diameter of all lymphomas at the site of injection. (TIF) [file pone.0034552.s001.tif]

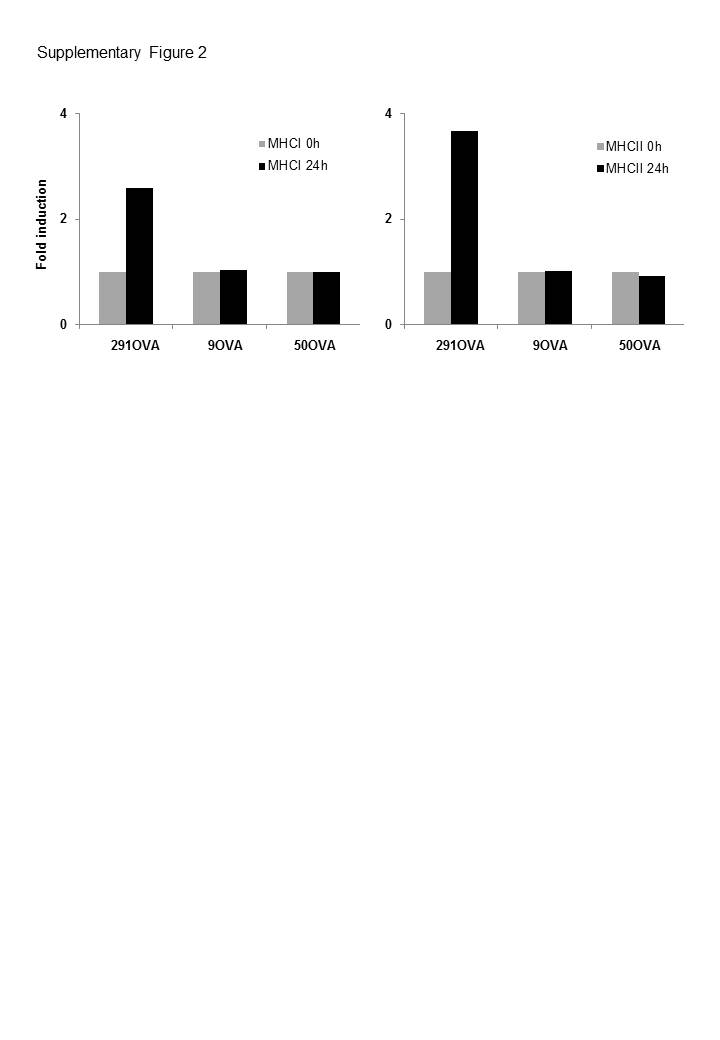

Supplement: Figure S2 — Induction of MHC class I and II by IFN-γ is dependent on STAT1- and IFN-γ receptor-signaling. 291OVA (wild-type), 9OVA (STAT1−/−), and 50OVA (IFN-γ-R−/−) were exposed to 100 U IFN-γ for 24 hours and MHC class I expression was assessed by flow cytometric analysis. STAT1-deficient and IFN-γ receptor-deficient lymphoma cells do not upregulate MHC class I upon IFN-γ treatment. (TIF) [file pone.0034552.s002.tif]
